# The Striatum and Subthalamic Nucleus as Independent and Collaborative Structures in Motor Control

**DOI:** 10.3389/fnsys.2016.00017

**Published:** 2016-03-01

**Authors:** Alia Tewari, Rachna Jog, Mandar S. Jog

**Affiliations:** London Health Sciences CentreLondon, ON, Canada

**Keywords:** striatum, subthalamic nucleus, motor control, cognition, basal ganglia

## Abstract

The striatum and the subthalamic nucleus (STN) are two separate input structures into the basal ganglia (BG). Accordingly, research to date has primarily focused on the distinct roles of these structures in motor control and cognition, often through investigation of Parkinson’s disease (PD). Both structures are divided into sensorimotor, associative, and limbic subdivisions based on cortical connectivity. The more recent discovery of the STN as an input structure into the BG drives comparison of these two structures and their respective roles in cognition and motor control. This review compares the role of the striatum and STN in motor response inhibition and execution, competing motor programs, feedback based learning, and response planning. Through comparison, it is found that the striatum and STN have highly independent roles in motor control but also collaborate in order to execute desired actions. There is also the possibility that inhibition or activation of one of these structures indirectly contributes to the function of other connected anatomical structures. Both structures contribute to selective motor response inhibition, which forms the basis of many tasks, but the STN additionally contributes to global inhibition through the hyperdirect pathway. Research is warranted on the functional connectivity of the network for inhibition involving the rIFG, preSMA, striatum, and STN.

## Introduction

The basal ganglia (BG) have been investigated as a control center for motor and cognitive behavior (Baláž et al., 2011) due to their dysfunction in Parkinson’s disease (PD; Hornykiewicz, 1966; Baláž et al., 2011). For many years, it was thought that the striatum was the only input structure of the BG but it is now known that the subthalamic nucleus (STN) is another entry point (Nambu et al., 1996; Baláz et al., 2012; Brunenberg et al., 2012).

Five cortico-BG-thalamo-cortical circuits have been identified: one motor, one limbic, two associative, and one occulomotor (Albin et al., 1989; Alexander et al., 1990). Within each circuit, there resides a direct, indirect, and hyperdirect pathway (Kitai and Deniau, 1981; Albin et al., 1989; Nambu et al., 1996; Baláz et al., 2012; Brunenberg et al., 2012). The majority of research has focused on the motor circuit due to its involvement in movement disorders such as PD. The direct pathway of this circuit is known to initiate movement and the indirect pathway to inhibit movement (Mink and Thach, 1991; DeLong and Wichmann, 2007). Recently, fibers connecting various motor, somatosensory, and frontal cortices to the globus pallidus were identified, suggesting there may be an additional direct cortico-BG pathway (Milardi et al., 2015). Due to its relatively new discovery, the hyperdirect pathway’s function in the cortico-pallidal pathway is still being investigated.

**Figure 1:**
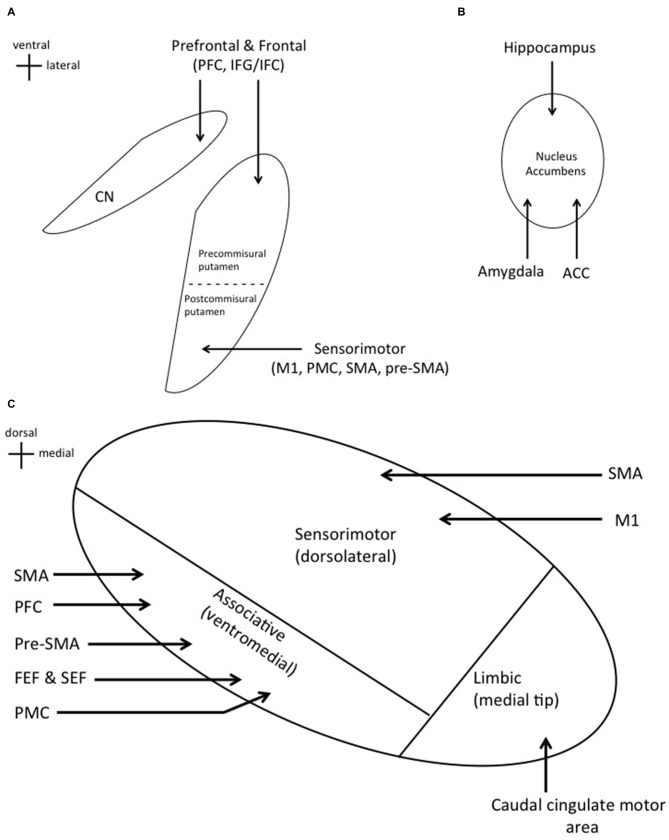
**(A)** Cortical inputs into specific regions of the dorsal striatum. Prefrontal and frontal cortical areas project to the caudate nucleus (CN) and precommisural putamen. Sensorimotor cortices project to the postcommisural putamen. **(B)** Projections from the hippocampus, amygdala, and anterior cingulate cortex (ACC) to the nucleus accumbens (NA; ventral striatum). **(C)** Cortical inputs into specific regions of the subthalamic nucleus (STN). Significant overlap is seen with these two input structures as expected.

The dorsal striatum is composed of the putamen and caudate nucleus (CN) and the ventral striatum of the nucleus accumbens (NA; Percheron et al., 1994; Afifi, 2003). Accordingly, the striatum is divided into functional subdivisions. The sensorimotor striatum is composed of the postcommisural putamen which regulates motor movements and receives projections from sensorimotor cortices (Selemon and Goldman-Rakic, 1995; Afifi, 2003). The precommisural putamen and CN (dorsal striatum) receive projections from prefrontal and frontal cortices, and together make up the associative striatum (Afifi, 2003; Phillips and Everling, 2012; Jarbo and Verstynen, 2015). The associative striatum is implicated in reasoning, planning, motor sequence learning, voluntary motor selection, and performance monitoring (Everitt et al., 1991; Cardinal et al., 2002; Jankowski et al., 2009). The limbic striatum is composed of the NA (ventral striatum) which receives projections from the anterior cingulate cortex (ACC), amygdala, and hippocampus, and is responsible for reward and motivation (Figures 1A,B; Afifi, 2003; Adler et al., 2013).

The STN is divided into similar functional subdivisions based on cortical projections. The dorsolateral STN houses the sensorimotor region, which receives direct projections from the primary motor cortex (M1) and supplementary motor area (SMA; Monakow et al., 1978; Parent and Hazrati, 1995; Romanelli et al., 2004). The ventromedial STN contains the associative region, receiving projections from the premotor cortex (PMC), prefrontal cortex (PFC), pre-SMA, SMA, frontal eye field (FEF), and supplementary eye field (SEF; Parent and Hazrati, 1995; Aron et al., 2007). Lastly, the medial tip represents the limbic region, which receives projections from the caudal cingulate motor area (Figure 1C; Takada et al., 2001).

With respect to the cellular structure, majority of striatal neurons are GABAergic medium spiny neurons (MSN; Gerfen and Young, 1988). MSN containing D1 receptors project to the internal globus pallidus (GPi) and SNr and give rise to the direct pathway (Gerfen et al., 1990). D2 containing MSN project to the external globus pallidus (GPe) and give rise to the indirect pathway (Gerfen et al., 1990). In addition, the striatum contains GABAergic and cholinergic interneurons (Kimura et al., 1980; Bolam et al., 1983). Striatal interneurons are known to receive projections from the cortex and thalamus, and may alter the activity of spiny projection neurons (Kawaguchi et al., 1995; Kawaguchi, 1997). The STN contains glutamatergic projection neurons and no interneurons (Nauta and Cole, 1978). The cyto-architectural connectivity within the striatum allows it to integrate multiple sources of information including information coming from the cortex, substantia nigra, thalamus and its intrinsic interneurons. The MSN of the striatum thus have a large dynamic range in terms of processing ability. Since the current understanding of information processing within the striatum suggests multiple distributed networks, the modulation and transfer of cortical information within the striatum is likely to be performed by many networks. Significant research regarding such network level information processing has been done for the striatal system, especially using novel electrophysiological tetrode technology. Habit learning may be a result of such distributed learning within the striatum (Jog et al., 1999). Jog et al. (1999) have also shown that the striatum may be involved in a method of information processing that allows a rapid and exponential growth in information computing that may be based upon its unique architecture (Aur and Jog, 2006; Jog et al., 2007; Aur et al., 2011).

In comparison, the method of information processing within the STN is relatively simpler but less well-understood. However, it is likely that the intrinsic properties of the neurons determine this processing ability and potentially have a lower dynamic range than the striatal neurons. Complex patterns of intrinsic activity arise in the subthalamic neurons, where extrinsic excitation likely controls timing of action potentials (Wilson and Bevan, 2011).

The discovery of two separate input structures of the BG compels us to question their individual and combined purposes. In this review, we aim to compare and contrast the functions of the striatum and STN in several domains. Specifically, we have summarized and discussed the roles of these two nuclei and their connections in terms of the currently explored behavioral roles. We have touched upon: (a) motor response inhibition; (b) competing motor programs; (c) performance monitoring and feedback; and (d) motor response planning and execution. In each section, we have also indicated potential new and future directions of research that may guide further The mechanistic underpinnings of the behavioral aspects of functional comparisons between the striatum and the STN are largely hypothetical and therefore beyond the scope of this article.

## Motor Response inhibition

### General Comments

The performance of a motor task which is likely coordinated through the fronto-basal ganglionic system requires appropriate action selection as well as motor response inhibition. The concept of inhibition is crucial as once an internally or externally cued action selection has occurred, the response has to be further modulated and regulated. Response inhibition is often investigated as selective vs. global inhibition and proactive (internally cued) vs. reactive (externally cued) inhibition (Mink, 1996; Ballanger et al., 2009; Smittenaar et al., 2013). Global inhibition is a reaction to a signal to stop all motor response. Selective inhibition defines the cessation of a particular component of a task so that the entire motor execution is not terminated and goal directed movement may be possible. This motor task cessation or inhibition could be preplanned or proactive, prior to the actual commencement of the task so that at the opportune moment. Such proactive inhibitory signal can be selective or global. In the selective mode only the unwanted sub-components of the task are stopped while others might continue on, whereas in the global mode all aspects are stopped. Reactive inhibition may occur to an external cue causing certain aspects of a motor task to be completed when potentially an unexpected external input is the driver. In this scenario, no prior information exists regarding the need to stop some or all components of the task. Again, this reactive, unplanned cessation can be to selected components of the motor task which is termed selective or reactive inhibition. Since this inhibition is “selective” it may actually lead to interference to task performance. Similarly, reactive global inhibition may result in completely stopping the task itself. One can find many examples of these phenomena in daily life where a full or partial brake is put on what we are doing either with knowledge such as slowing down to open a door (selective/proactive) or modifying a reach or trajectory when an unplanned obstacle approaches (selective/reactive). In similitude, stopping at a red light while driving (global/proactive) vs. when stopping when someone unexpectedly opens the door (global/reactive) are examples of global motor response inhibition.

The BG through their connectivity and anatomical architecture are felt to be major contributors to this balance. The anatomical systems are of course not mutually exclusive and hence information is very likely to be shared across the hierarchically connected structures (Aron and Poldrack, 2006; Jahfari et al., 2011; Toxopeus et al., 2012; Schel et al., 2014). In order to strike this balance, a system of direct and reciprocally indirect pathways of connections appears to exist in the major nuclei including the striatum, STN and now even the pallidum as reviewed briefly above. Both the indirect and hyperdirect pathways play a significant role in response inhibition (Aron and Poldrack, 2006; Jahfari et al., 2011; Toxopeus et al., 2012; Schel et al., 2014). The striatum and STN are thought to execute proactive selective inhibition through the indirect pathway which is common to the STN and the striatum (Smittenaar et al., 2013). The STN has been found to also be involved in reactive global inhibition through the hyperdirect path (Vink et al., 2005; Zandbelt et al., 2013b). Since the STN is the further downstream structure and receives direct input from the cortex, it is not surprising that it has been implicated in the fast and global inhibitory role (Jahfari et al., 2011; Smittenaar et al., 2013), The anatomy of these pathways supports these findings, with the hyperdirect pathway controlling global inhibition and the indirect pathway executing selective inhibition (Majid et al., 2013; Smittenaar et al., 2013). In comparison, proactive vs. reactive inhibitory differences in terms of anatomical control are less clear (Coxon et al., 2007, 2009; Aron and Verbruggen, 2008). Proactive inhibition is more likely to produce less interference to task performance as it is pre-planned while reactive inhibition is less selective and interfering. In fact, direct cortical control may be involved in terms of such a balance between reactive vs. proactive planned inhibition. Preliminary data shows that a balance may exist then between the speed in the inhibition vs. selectivity, hence the faster the inhibition, the less selective it becomes. In this framework, one can envision that the cortical-striatal-pallidal pathway would inherently be slower and so would be involved in the selective and proactive inhibition while the cortico-subthalamic system would be more likely to be global and probably reactive (Smittenaar et al., 2013).

PD patients undergoing STN stimulation have shown increased impulsive behavior. This release phenomenon presumably based upon the inactivation of the STN from the BG loop may be indicative of the fact that the STN is indeed the primary source for reactive global inhibition (Frank, 2006; Frank et al., 2007; Cavanagh et al., 2011; Green et al., 2013) and its deactivation results in a loss of global impulse control. Similarly, impairment of executive cortical control has been hypothesized to result in the loss of inhibitory behavioral control leading to impaired habit formation, addiction and even attention deficit disorders and motor tics (Isoda and Hikosaka, 2011; Ersche et al., 2012).

### Striatum

The striatum’s role in response inhibition has been most commonly studied using fMRI coupled to behavioral tasks such as the Stop Signal task and the Go/NoGo task. Successful stop trials have shown bilateral putaminal activation, along with sensory motor cortex, right inferior frontal cortex (rIFC), ACC, and bilateral parietal cortical activation (Vink et al., 2005; Zandbelt and Vink, 2010; Schel et al., 2014). These regions, along with the SMA, pre-SMA, inferior frontal gyrus (IFG), and dorsolateral PFC (DLPFC), have also shown increased activity during the preparatory phase of response inhibition, suggestive of this network’s involvement in proactive inhibition (Vink et al., 2005; Zandbelt and Vink, 2010; Smittenaar et al., 2013; Zandbelt et al., 2013b). Go/NoGo tasks have shown functional connectivity between the rIFC and the ventral striatum, where successful inhibition led to activation in the rIFG and decreased activity in the ventral striatum (Behan et al., 2015). In a study by Jahfari et al. (2011), “fast inhibitor” participants with shorter stop-signal reaction times (SSRTs) showed increased connectivity between the rIFG and right caudate, whereas the “slow inhibitor” participants with a longer SSRTs showed increased connectivity between the pre-SMA and right caudate. Additionally, successful inhibition is associated with M1 deactivation, where the degree of deactivation is proportional to the degree of striatal activation (Zandbelt and Vink, 2010; Vink et al., 2013).

Conflicting evidence arises with regards to the activity of the putamen, CN, and IFG/IFC during such tasks. Many studies have found the putamen to be active during selective proactive inhibition as it is a major nucleus in the BG motor loop (Schmidtke et al., 2002; Smittenaar et al., 2013). However, other studies have found CN activation related to inhibition (Jahfari et al., 2011; Majid et al., 2013). Additionally, although activity in the IFC/IFG has been reported, TMS studies have shown rIFC stimulation to have no effect on proactive inhibition (Verbruggen et al., 2010; Zandbelt et al., 2013a).

### Sub-Thalamic Nucleus

Reactive and global inhibition through the hyperdirect pathway ellicit activation in similar cortical areas. Parallel pathways projecting from the IFG/IFC and the pre-SMA to the STN have been proposed, along with a hierarchal pathway from the pre-SMA through the rIFG to the STN (Aron and Poldrack, 2006; Aron et al., 2007; Ballanger et al., 2009; Fleming et al., 2010; Forstmann et al., 2010; Neubert et al., 2010; Wiecki and Frank, 2010; Coxon et al., 2012; Herz et al., 2014; Rae et al., 2015). The Stop Signal task in association with fMRI has shown the rIFC to directly excite the STN in successful stop trials (Aron and Poldrack, 2006). A more recent study has shown a positive correlation between efficiency of stopping with tract strength (voxel values proportions) of both IFG-STN and pre-SMA-STN pathways (Rae et al., 2015). However, greater activity was found in the IFG-STN pathway when pre-SMA activity was disrupted (Herz et al., 2014). Thus, the parallel pathways may work independently or complement each other during response inhibition (Wiecki and Frank, 2010; Herz et al., 2014). The hierarchal pathway shows increased functional connectivity between the pre-SMA and the rIFG, where activity in the pre-SMA occurs earlier than the IFG during action inhibition (Duann et al., 2009; Neubert et al., 2010; Herz et al., 2014).

### Future Research Questions and Directions

The striatum and STN may have more in common than just being input structures of the BG. As nuclei of the indirect pathway, the striatum and STN work together for selective inhibition. The hyperdirect pathway extends the STN’s function to global inhibition as well. The current understanding and the unmet needs in investigating motor performance in terms of the comparison of the cortical and BG structures is summarized in Table 1.

**Table 1:** **Summary of motor inhibition and the possible subcomponents including anatomical connectivity**.

	Proactive	Reactive
Selective	• Preparatory phase/planning • Selective and less interference • Balance between accuracy and speed • Striatum/Pallidum are involved • Cortical involvement	• No preparation/planning • Not-accurate, but maybe selective • Interference with ongoing motor tasks • Possibly striatal/pallidal? • No cortical involvement?
Global	• Preparatory phase/planning • Global motor inhibition • Anatomical control largely unknown but STN may be involved	• No preparatory phase • General stopping of movement • Possibly subthalamic • No cortical involvement • STN-descending brain stemtract involvement

As one example of the global questions that still need to answered, research identifying the hyperdirect pathway’s involvement in proactive global inhibition has not been done. Only two studies have been conducted and concluded that it has not been possible to study the inhibition of the motor system globally when given preparatory signals (proactive inhibition; Aron and Verbruggen, 2008; Greenhouse et al., 2012). The study of such proactive global inhibition is therefore an unmet need. To some extent the global motor function cessation would be difficult to image as it would probably require mobile imaging techniques. Numerous research questions remain open at this point for motor inhibition and the role played by the cortical-BG systems:

1. Are there preferential areas of the striatum, especially putamen that are preferentially activated or connected to specific cortical anatomical structures functionally?
2. How do these subsets of connectivity relate to the proactive vs. reactive inhibition? What are the relationships to the nature of tasks that we are expected to perform?
3. Is there a difference between limb vs. whole body motor tasks such as gait?
4. What are the ways in which ecologically valid tasks such as what we do in our day to day living be incorporated in studying these relationships?

Many of the above questions need to be investigated in animal models as it is not possible to study multi-site anatomical connectivity within humans. In the human, two new technological advances have recently been developed that show promise in investigating cortical and deep brain structures. Recently, ambulatory functional near infra-red spectroscopy (fNIRS) have been successfully piloted and tasks that explore global inhibition in an ambulatory state can potentially now be investigated (Piper et al., 2014). However, these recordings are restricted to the cortical surface. The ability to perform ambulatory recordings while having implanted electrodes in the STN and in some cases simultaneously in the cortex with subdural electrodes or ambulatory electroencephalography chronically in ambulatory patients is another powerful way to study global inhibition (Shimamoto et al., 2013). This electrophysiological setup can be combined with the fNIRS technology while designing and studying proactive global inhibition.

## Competing Motor Programs

### General Concepts: Response Threshold, Speed-Accuracy Tradeoff and Laterality

Having considered inhibition, we now turn to understanding the role of the striatum and STN in allowing action to be performed. At any given time, many motor actions compete for expression (Frank, 2006). The selection and execution of the most appropriate response while tailoring inhibition among others is one application of response inhibition. To allow for the expression of a proper motor action, the direct, indirect, and hyperdirect pathways have been conjectured to work together in terms of enabling action (Frank, 2006).

Three key concepts in decision-making are response thresholds, speed-accuracy trade-offs and conflict resolution. Response thresholds represent the amount of information required before a decision can be made (Forstmann et al., 2008). A higher response threshold allows for “accumulation” of information within a structure before neurons within that structure respond. It is currently thought that the STN increases response thresholds to inhibit impulsive actions (the so-called “hold-your-horses” response) while the striatum decreases response thresholds through the indirect pathway for the opposite effect (Frank, 2006; Frank et al., 2007; Mansfield et al., 2011; Obeso et al., 2014). The response thresholds are modulated based on whether speed or accuracy is to be favored in the task at hand (Frank, 2006; Frank et al., 2007; Obeso et al., 2014). Finally, behavioral conflict resolution which may involve similar motor response also has to occur to preferentially select a behavior (e.g., buy coffee or a sandwich with the same amount of money). This construct is similar to the one discussed above in terms of the selective inhibition of subcomponents of tasks.

Studies employing fMRI have shown the pre-SMA to modulate the striatum’s response in threshold setting (Forstmann et al., 2008; Mansfield et al., 2011). Repeat cues indicating that the same task is to be performed result in greater activation of the pre-SMA and bilateral striatum compared to switch cues. This finding is in accordance with the view that the striatum decreases response threshold, as repetitive actions do not require additional information (Mansfield et al., 2011). Trials in which speed is favored have shown activation of the pre-SMA and anterior striatum, leading to a decreased response threshold and a faster reaction time (Forstmann et al., 2008). However, when accuracy is favored, increased IFG-STN activity has been identified (Herz et al., 2014). This has been further investigated by comparing patients that have undergone subthalamotomy surgery. When compared to unoperated patients or control group, PD patients who had undergone unilateral subthalamotomy showed contralateral abnormality in the speed-accuracy tradeoff in a Go reaction time paradigm. Although the speed improved to the level of controls in this task, the accuracy significantly deteriorated, implying that lesions in the STN impair regulation of selective accuracy of the task. This difference was especially observed with right sided subthalamotomy when compared to the left. Therefore, patients showed higher speed but higher errors in the task with right sided surgery (Obeso et al., 2014). Patients with right subthalamotomy showed no proactive inhibition with the contralesional hand. These studies indicate that the right sided STN network is more likely to be involved in action inhibition after selection and is a combination of the preSMA, iFG, probably caudate and the STN. The right sided system may be involved in bilateral control. However, the basis for this difference is unclear and no data exists as to the differences in the connectivity between the right vs. left cortico-basal ganglionic networks and indeed the reason for such localization remains a mystery (Garavan et al., 1999; Obeso et al., 2013).

### Currently Proposed Model

Frank et al., present a neurocomputational model for decision making which brings together the direct, indirect and hyperdirect pathways in a three-stage process. When presented with a stimulus, multiple motor responses generated in the PMC activate the striatum. The striatum attempts to execute a premature response through the direct pathway. At the same time, the indirect and hyperdirect pathway activate the STN, which prevents inhibition of the GPi from the direct pathway. As a result, thalamic inhibition leads to global inhibition of all competing motor responses, increasing the response threshold which slows the task performance and reduces impulsivity. Once enough information has been collected and a decision has been made, both decreased cortical activity and the reciprocal interconnections between the STN and GPe decrease STN activity. The striatum, through the direct pathway, inhibits specific GPi columns, which allows the thalamus to excite M1 resulting in the execution of the chosen motor response. Once this occurs, the STN is again activated through the indirect pathway to terminate the response (Frank, 2006). The decision making process is a good example of how the striatum and STN use their distinct functions in conjunction to perform a task (Table 2). However, this model does not take into account the laterality of responses as discussed above or the breadth of role of cortical connectivity to these structures.

**Table 2:** **A summary of the similarities and differences between the striatum and subthalamic nucleus (STN) in four different tasks: motor response inhibition; competing motor programs; performance monitoring and feedback; and motor response planning and execution**.

Motor response inhibition		
Similarities	Differences	
	
	Striatum	STN
• Activation of IFC and pre-SMA during inhibition	• Selective and proactive inhibition via indirect pathway • Slow inhibition	• Global and reactive inhibition via hyperdirect pathway • Fast inhibition

**Competing motor responses**		

• Involvement of indirect pathway for inhibition of actions	• Decrease response threshold • Favor speed over accuracy • Dual function: (i) fast premature response via direct pathway and (ii) contribute to inhibition via indirect pathway	• Increase response threshold • Favor accuracy over speed • Single function: fast inhibition via hyperdirect (and indirect) pathway

**Feedback based learning**		

• Both contribute to performance monitoring and feedback	• Via associative and limbic circuit • Dopamine plays a role in feedback	• Via the degree of reduction of beta band activity • Post-error slowing

**Motor preparation and execution**		

• Influence saccades • Project to SC	• Direct pathway to execute movement • Sensorimotor striatum active for habitual tasks; associative striatum active for novel tasks • Generate saccades	• Contributes indirectly by decreasing beta power and through suppression of beta oscillations • Suppress automatic saccades; activate controlled saccades

*Abbreviations: IFC, inferior frontal cortex; pre-SMA, pre-supplementary motor area; SC, superior colliculus*.

### Future Research Questions and Directions

An important unmet need is the lack of understanding of the anatomico-functional connectivity differences between the right vs. the left sided networks. Human imaging studies have not been able to elucidate these differences. Similarly, behavioral tasks have been limited to the go-no-go, go-stop or similar tasks. In order to address these questions, several lines of research could be suggested:

1. Lesional studies in animal models is an important method by which specific pathways could be studied. The lack of specific lesions in humans in the regions of interest may be resolved this way. Targeted anatomical and electrophysiological studies have been done over the years. However, with the improved understanding of the specific lateralized pathways, such studies should now be repeated with comparison between right vs. left sided networks.
2. Similarly, patients that have had BG strokes specifically in the regions such as the STN or pallidum are a niche population that are relatively common. These patients could be studied behaviorally as well as with imaging. The study of this population is currently lacking.

## Feedback Based Learning

### General Comments: Reinforcement and Error Driven Learning

The constructs of action selection including inhibition have been discussed. Once performed, a critical component of task performance is monitoring of the action being performed, especially to modify the next action set. The entire BG complex is involved in motor learning and the execution of movements (Wu et al., 2004). Performance monitoring and feedback are important aspects of any motor action as they allow for the adjustment of subsequent actions. During movement, the rostral ACC seems to be activated by the frontal cortex and BG, sending information to motor areas for modification of the task set. This modified information is fed back to the frontal cortex and BG to be applied in upcoming actions (Holroyd and Coles, 2002; Schel et al., 2014). The associative circuit (involving the frontal cortex, CN and anterior putamen) has been shown to modulate performance monitoring and feedback (Hikosaka et al., 2002; Jankowski et al., 2009; Yamada et al., 2011). Studies using fMRI have found the putamen’s mechanism of feedback-based learning to be one of stimulus-action-reward associations, compared to the CN’ feedback which is based on actual vs. predicted rewards (Haruno and Kawato, 2006).

### Striatum

An important aspect of such feedback learning has been investigated extensively with respect to the role of the striatum. Reward prediction, action strategy and the rules of action performance are behavioral subcomponents that are felt to be important in the monitoring of action performance. The striatum is known to be proactively involved in this by allowing a comparison between estimated value of a predicted reward to the action being performed and the strategy that is being employed to do the task. Due to the convergence of input into the striatum from large cortical areas, the ventral and dorsal striatum has been studied as the center where the value and strategy balance is felt to be decided. Electrophyisiological striatal recordings from macaques during numerous trial-and-error, reward driven tasks have shown that the striatum is indeed involved in the proactive and selective decision making and neurons can encode both positive and negative results of action selection. Interestingly, striatal neuronal firing patterns persisted and remained stable till the action was completed and presumably the cortico-striatal feedback was received, especially from the PFC (Haber et al., 2006). Finally during adaptive/proactive learning process striatal neurons have been shown to electrohysiologically encode the strategic adaption of action selection and performance, representing a reinforcement based, slow, proactive strategic learning (Pasquereau et al., 2007; Lau and Glimcher, 2007, 2008).

The cortico-striatal connectivity with the ventral striatum is especially interesting with its association to the limbic circuit and is also thought to contribute to performance monitoring and feedback-based learning (Haruno and Kawato, 2006; Valentin and O’Doherty, 2009; Simões-Franklin et al., 2010; Wilkinson et al., 2014). The DLPFC in conjunction with the NA showed increased neuronal firing during errors made in a Go/NoGo task (Simões-Franklin et al., 2010). Like the CN, the NA provides feedback by comparing actual and predicted rewards (Haruno and Kawato, 2006). Finally, the role of dopamine has been investigated extensively in enabling the reinforcement learning seen in the striatum. A recent study using PET scans has shown dopamine to modulate feedback-based learning, where the ventral striatum increases dopamine release associated with feedback (Wilkinson et al., 2014). In similitude, electrophysiological striatal recordings have identified another component of the limbic circuit, the ACC, in action evaluation and error detection (Carter et al., 1998; Yamada et al., 2011).

### Subthalamic Nucleus

The role of the STN in the monitoring of ongoing activity during action performance has also been investigated. Such intra-action motor adaptation has been usually associated with cerebellar modulation (Tseng et al., 2007; Miall and King, 2008). However, the role of the cortical and subthalamic correlation in action modulation has been advanced by intraoperative human recordings. Luft et al. (2014) have shown that beta oscillation (13–30 Hz) suppression occurs over the sensorimotor cortex during error feedback processing which may represent cortical reorganization during action correction and subsequent motor learning. The STN shares this ability to monitor ongoing motor performance and identify error. In a joy-stick task performed during simultaneous cortical EEG and STN LFP in PD patients Tan et al. (2014) showed that the degree of error in movement is reflected in the degree of reduction of beta band synchronization within the STN. Event-related potentials in the STN appear 260–450 ms after an error, suggesting the STN processes error and sends this information to the sensorimotor cortex for task revision (Siegert et al., 2014; Tan et al., 2014). The calculation of the phase/amplitude of occurrence of this activity coupling also showed that the flow of information in this system from the STN to the cortex increased with larger error and adjustment/correction of movement in subsequent tasks. Unlike the striatum, the STN also contributes to post-error slowing (Rabbitt, 1966; Cavanagh et al., 2014). Through the hyperdirect pathway, the STN’s 2.5–5 Hz phase activity is enhanced, leading to increased response thresholds and a slower response (Dutilh et al., 2012; Cavanagh et al., 2014). As such the error correction represented by the cortico-STN-pallidal-cortical system is much more likely to be reactive and non-specific (global) rather than what is done through the striatal reinforcement learning system. A significant limitation of this work is the lack of control data as the subjects studied all had PD, were on dopaminergic medication and had large implanted electrodes that may have produced acute lesional changes.

As an application of response inhibition, the striatum’s contribution to post-error slowing is something to be investigated. Having two structures within the BG performing the same function seems unlikely, and thus there exists the possibility that the STN and striatum modulate each other to monitor the ongoing performance. Behavioral and electrophysiological investigations that monitor chronic activity in an *in vivo* model while data is gathered from cortex/striatum and the STN simultaneously is an approach that could shed light on these mechanisms (Table 2). Thus, it is likely that the striatum though the direct pathway enables an action and then through the indirect pathway continues to monitor and adjust proactive and selective inhibition. Such error-prediction related learning is then further reinforced through the dopaminergic signal from the midbrain (Bayer and Glimcher, 2005; Tobler et al., 2005). On the other hand, the cortico-subthalamic system may be involved in the early and more global pro and reactive learning by making larger corrections/predictions by its connections to the cortical networks.

### Future Research Questions and Directions

The ability to now record in humans intraoperatively is an enormous advance. Until now, such recordings as discussed briefly above have been restricted to acute intraoperative or immediate post-operative periods. The intrinsic difficulties with post-surgical effects on networks have limited the interpretation and significance of the data such as the oscillatory behavior seen in many neuronal structures. Similarly, the electrode configurations have only allowed recordings from one or at most two contact points. The improvement in technology for electrodes, the ability to record within the implanted pulse generator and the ability to chronically record from implanted electrodes in humans are all advances within the past 3–5 years. Based upon these, our laboratory along with others have embarked upon the collection of human behavioral and electrophysiological data from a variety of patients under acute and chronic settings:

1. Multi-site sub-dural cortical and BG depth electrode recordings intraoperatively in patients with PD and dystonia undergoing standard accepted behavioral tasks such as go-no-go and response inhibition, among others.
2. Chronic long-term EEG and deep brain field potential recordings in fully ambulatory patients using the “brain-radio” system of recording in the same disorders.
3. Control patient sub-dural cortical data collection in patients undergoing cortical mapping for epilepsy using behavioral tasks similar to the above experiments.

## Motor Preparation and Execution

The constructs of motor planning, execution including the concepts of inhibition, error correction and reinforcement have all been discussed in some detail above. The direct, indirect and hyperdirect pathways along with certain areas of the cortex, STN and striatum and even dopaminergic input from the substantia nigra are heavily involved in such reinforcement learning. In this model, the direct pathway has been felt to be responsible for the enablement of execution of actions and only involves the STN indirectly (DeLong and Wichmann, 2007; Wichmann and Dostrovsky, 2011). The sensorimotor striatum is critical in performance of habitual/automatic movements, while the associative striatum is involved in the acquisition of new motor skills and regulating goal-directed behavior (Miyachi et al., 1997). Using fMRI, Jankowski et al. (2009) identified bilateral activity in the associative striatum during the planning phase of a novel task. However, during the execution of this novel task, activity was shifted in a caudal manner to the sensorimotor striatum (Jankowski et al., 2009). In support of this finding in a raclopride binding PET study in humans, Lappin et al. (2009) showed that in sequence learning and spatial mapping tasks there is an increase in dopamine release in the associative striatum during planning, while dopamine levels in the sensorimotor striatum increased during active motor control. In opposition, during habitual tasks Wu et al. (2014) identified augmentation of putamen activity posteriorly and decreased activity anteriorly. Moreover, movement frequency—but not complexity—was associated with an increase in sensorimotor striatum signals in fMRI (Lehéricy et al., 2006). However, activity in the associative striatum was increased in association with movement frequency and complexity (Lehéricy et al., 2006).

As mentioned, the STN contributes to movement execution indirectly. Electrophysiological recordings show the classical decrease in STN beta power before and during movement (Oswal et al., 2013; Benis et al., 2014), with beta oscillation suppression during self and externally paced movement (Cassidy et al., 2002; Levy et al., 2002; Williams et al., 2005; Kühn et al., 2006). The timing of oscillation suppression has been shown to positively correlate with the movement-initiation reaction time (Kühn et al., 2006). Furthermore, task complexity correlates with beta desynchronization only if the parameters of the task can be prepared in advance (Oswal et al., 2013). The timing of movement-related potential onset in the STN correlates with pre-movement activity in the SMA and M1 (Paradiso et al., 2003). Through its deactivation, the hyperdirect pathway contributes to movement planning and execution (Paradiso et al., 2003).

In this section, we have presented the details on the striatum and STN’s contribution to movement execution. Thus, it is important to revisit the second phase of the decision making model presented by Frank (2006; execution of the selected action) as it represents how the striatum and STN work together (but in their own ways) to allow for movement execution (Table 2).

## Conclusion

This review has presented the motor task from the planning, performance, execution and inhibition perspectives, comparing the fronto-striatal vs. cortico-subthalamic controls. We have touched upon highlights including the current deficiencies and the possible new innovations that may help elucidate, especially in humans, mechanisms of global and complex motor control and action performance. The identification of dual input structures into the BG, with similar functional subdivisions, drives us to compare and contrast each structure. We have focused upon the similarities and differences between the two main input/output structures of the BG in terms of their contributions towards motor learning and motor behavior. These differences are based upon the differences between the anatomy, network connectivity and neurochemistry of these two structures. The striatum and the STN work together through the indirect pathway for selective response inhibition. Independently, the striatum promotes action execution via the direct pathway and the STN acts as a fast global response inhibitor through the hyperdirect pathway. The decision-making model presented by Frank (2006) portrays the independent and collaborative nature of the striatum and STN. From this model, it is evident that although they have different inherent functions, the inhibition of their respective functions allows for the indirect contribution to the other structure’s function.

## Future Directions

A significant amount of work remains to be done to further elucidate the contributions using novel and mainly human work. Some of this work using targeted multisite recordings has already begun in the last 5 years. Although not exhaustive, some aspects of the direction of this work in humans has been provided in every section as possible unmet needs.

It has been clearly established that the IFG/IFC and pre-SMA are significant neural structures working with the STN and striatum for response inhibition. The biggest question to be addressed is whether the functional connectivity of the network for inhibition, involving the rIFG and preSMA, is the same for the hyperdirect and indirect pathway. Novel behavioral tasks that specifically target the role of the STN during intraoperative recordings in patients continues to be an important avenue for determining the roles of the striatal vs. the subthalamic pathways for behavioral aspects of motor control. We have embarked upon this work in our laboratory in the operating room using behavioral tasks in patients undergoing STN and pallidal deep brain stimulation. In this set of experiments, simultaneous recordings are being carried out using electrodes over the cortical areas through the already existent burr holes. Field potential recordings via these subdural electroencephalography recordings are being collected while patients are performing behavioral tasks which include go/no-go as well as tasks that have been validated to test for action inhibition. At the same time, spike recordings are being made in the STN and in others in the pallidum to determine the relative differences between the recordings obtained through the pallidum vs. those from the STN.

Building upon the work by the Starr group (Shimamoto et al., 2013), correlational analysis between the field recordings from the cortex and the spike time dependent recordings from the STN and the pallidum will be performed in order to try to separate the relative contributions of these areas to the performance of the behavioral motor tasks. These studies are currently ongoing and recruitment is underway at our center to exactly study the controversies and contributions of the striato-pallidal pathways vs. the hyperdirect pathway through the STN. Pharmacological manipulation within the operating room has been done before with short acting medications such as apomorphine. However, another important avenue of exploration would involve using specific antagonists for glutamate and GABA within the structures themselves. Newer technologies that allow for intracerebral microinjection instruments (IMIs) to be placed along with the recording electrodes are now becoming available for use intraoperatively in humans (Bjarkam et al., 2010). Employing these technologies along with the multi-site recording methodologies will help further elucidate the neurochemical basis of these differences and what happens with excitation or inhibition, *in vivo*.

The ability to record from various brain structures while performing targeted behavioral and motor tasks in awake and behaving patients is also to some extent a reality. In another project in our laboratory, mobile recording technology using the Active PC+S DBS system (Gunduz et al., 2015) is being used to simultaneously record STN and pallidal signals while recording scalp EEG in awake and fully mobile patients, chronically. This project has just begun.

This type of behavioral tasks that are being employed in our studies, while recording from superficial and deep brain structures simultaneously will serve as extremely important framework to sorting out the contributions of various pathways. Intracerebral instrumentation is now becoming extremely routine in other conditions such as epilepsy. Such innovative use of intracerebral recordings along with pharmacological manipulation is the next future for understanding the roles played by structures such as the STN and the striato-pallidal circuits in motor learning and behavior.

## Author Contributions

All authors listed, have made substantial, direct and intellectual contribution to the work, and approved it for publication.

## Conflict of Interest Statement

The authors declare that the research was conducted in the absence of any commercial or financial relationships that could be construed as a potential conflict of interest.
